# More Than Three Years of Hepatic Recurrence-Free Survival After Radiofrequency Ablation for Hepatic Metastasis From Triple-Negative Breast Cancer

**DOI:** 10.7759/cureus.88578

**Published:** 2025-07-23

**Authors:** Bunzo Nakata, Chie Sakimura, Masashige Tendo, Hideaki Yokomatsu, Takeshi Hori

**Affiliations:** 1 Surgery, Kashiwara Municipal Hospital, Kashiwara, JPN; 2 Surgery, Moriguchi Ikuno Memorial Hospital, Moriguchi, JPN

**Keywords:** bone metastasis, chemotherapy, hepatic metastasis, radiation therapy, radiofrequency ablation, triple-negative breast cancer

## Abstract

The prognosis of triple-negative breast cancer (TNBC) with hepatic metastasis is generally poor. Here, we report the case of a 70-year-old woman with TNBC whose hepatic metastasis did not recur for more than three years after radiofrequency ablation (RFA). During this period, the patient received chemotherapies with relatively mild side effects, such as eribulin mesylate and gemcitabine, maintaining a good quality of life. Although PARP inhibitors and immune checkpoint inhibitors are now available for TNBC, many cases are not eligible for these treatments due to the absence of specific target molecules. RFA may be a useful option in selected cases of hepatic metastasis from TNBC.

## Introduction

Patients with triple-negative breast cancer (TNBC) have a higher rate of distant recurrence and a poorer prognosis compared to other breast cancer subtypes. Fewer than 30% of women with metastatic TNBC survive beyond five years [1]. Due to the lack of targetable receptors, estrogen receptor, progesterone receptor, and human epidermal growth factor receptor 2 (HER2), treatment options for TNBC are limited [2]. Liver metastasis has been reported as the initial site of recurrence in approximately 13.7% of TNBC cases [3]. While hepatic metastases from breast cancer are typically regarded as a manifestation of systemic disease with a poor outlook, radiofrequency ablation (RFA) has shown promise as a safe treatment option in selected patients, potentially contributing to prolonged survival [4,5]. Here, we report a case of hepatic metastasis from TNBC that did not recur for over three years following RFA treatment.

## Case presentation

A 70-year-old postmenopausal woman presented to our hospital in September 2017 with a palpable mass approximately 4 cm in diameter near the right nipple. Core needle biopsy of the breast lesion revealed invasive ductal carcinoma. Preoperative staging, including chest plain computed tomography (CT), contrast-enhanced abdominal CT, and bone scintigraphy, showed no evidence of distant metastasis. Pathological examination of three sentinel lymph nodes confirmed extensive metastatic involvement. The patient underwent total mastectomy with axillary lymph node dissection. Fourteen out of 26 dissected lymph nodes, including all sentinel nodes, were positive for metastases. Immunohistochemistry confirmed triple-negative breast cancer (TNBC), and the cancer was staged as pT2N3aM0, corresponding to pathological Stage IIIC. Adjuvant chemotherapy was initiated with adriamycin + cyclophosphamide (AC), followed by tri-weekly paclitaxel. However, AC was discontinued after one cycle due to severe ischemic colitis. Post-chemotherapy, adjuvant radiotherapy was delivered to the right chest wall and right supraclavicular region.

**Figure 1:**
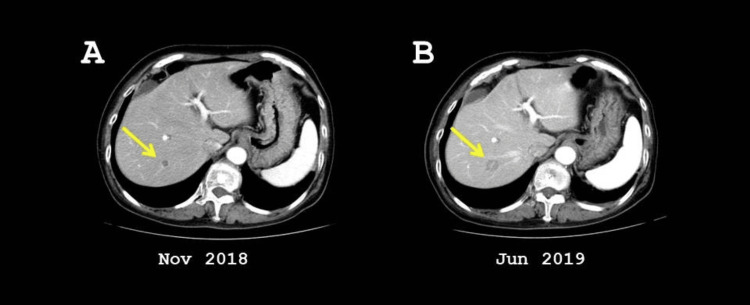
Abdominal CT images prior to RFA. (A) A hepatic metastasis in segment 7 from triple-negative breast cancer was identified in November 2018 (yellow arrow). (B) Despite chemotherapy with paclitaxel plus bevacizumab, the lesion increased in size by June 2019. CT: computed tomography; RFA: radiofrequency ablation.

In November 2018, follow-up contrast-enhanced abdominal CT detected a solitary hepatic metastasis measuring 11 mm in segment 7 (Figure 1). Consequently, the patient was treated with weekly paclitaxel plus bevacizumab. By June 2019, repeat imaging showed progression of the hepatic lesion, which had enlarged to 15 mm in diameter.

**Figure 2:**
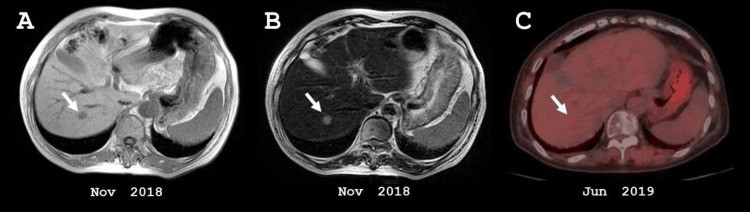
Abdominal MRI and PET-CT before RFA. (A) T1-weighted MRI. (B) T2-weighted MRI. (C) PET-CT showing 18F-FDG uptake (SUVmax = 2.54) in the hepatic metastasis in segment 7 (white arrow). MRI: magnetic resonance imaging, PET: positron emission tomography, SUV: standardized uptake value, FDG: fluorodeoxyglucose.

No other metastases were detected apart from the known solitary hepatic lesion, as confirmed by abdominal and chest CT, abdominal magnetic resonance imaging (MRI), and 18F-fluorodeoxyglucose (FDG) PET-CT (Figure 2). RFA was performed in July 2019.

**Figure 3:**
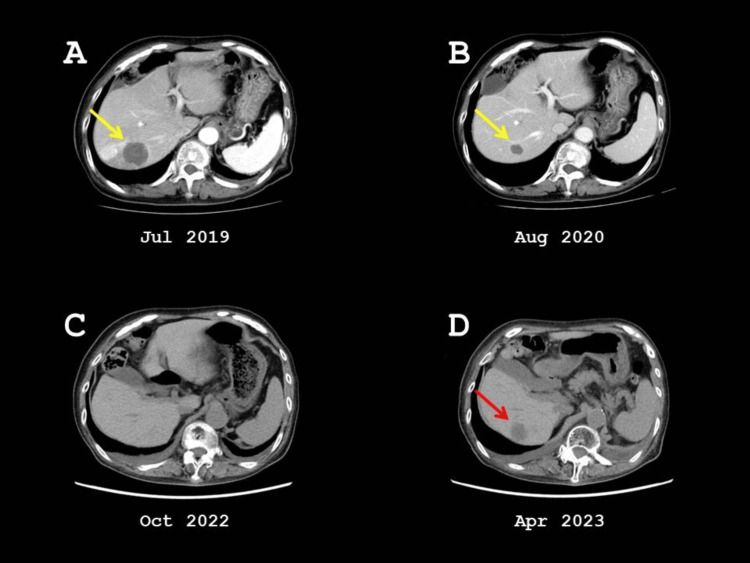
CT images after RFA. (A, B) The necrotic hepatic metastasis in segment 7 (yellow arrow) gradually decreased in size following RFA and eventually disappeared. (C) No new hepatic metastasis was observed until October 2022. (D) A new hepatic metastasis in segment 6 (red arrow) was detected in April 2023. (A) and (B) are enhanced CT scans; (C) and (D) are plain CT scans.

Under conscious sedation with 15 mg of pentazocine and local anesthesia with xylocaine, a Cool-tip RF needle (17 gauge, 20 cm length, 2 cm tip) was inserted through the right intercostal space to the hepatic metastasis under contrast-enhanced ultrasonography guidance. Track cauterization was performed using a Cool-tip RF generator, allowing the tip temperatures to reach 80℃. Follow-up enhanced CT scans after RFA showed gradual shrinkage of the ablated necrotic tissue, with no evidence of recurrent hepatic metastasis up to October 2022 (Figure 3).

Eribulin mesilate was administered after RFA, as it is indicated for patients with recurrent breast cancer previously treated with regimens including an anthracycline and a taxane [6]. In December 2021, bone metastasis to the third lumbar vertebra was treated with radiotherapy (30 Gy in 10 fractions). Gemcitabine was subsequently given to manage eribulin-resistant bone metastasis [7]. In April 2023, CT imaging revealed a new hepatic metastasis measuring 30 mm in segment 6 (Figure 3). This lesion was treated with RFA in May 2023. However, two additional hepatic metastases in segment 1 were detected one month later. In July 2023, new bone metastases involving the 6th to 9th thoracic vertebrae were irradiated with 20 Gy in five fractions. At that time, no recurrence was observed in the lungs or lymph nodes. S-1, an oral chemotherapeutic agent, was introduced for gemcitabine-resistant metastatic disease [8]. The patient passed away at home in February 2024 due to progression of her primary disease, breast cancer.

## Discussion

The median survival of untreated breast cancer patients with hepatic metastasis is reported to be as short as 4-8 months [9]. Currently, systemic chemotherapy remains the standard treatment for hepatic metastasis from breast cancer. In selected cases, hepatic resection and/or RFA have been performed, with relatively few adverse events and low mortality [10]. A recent case-control study demonstrated that while hepatic resection and/or RFA for isolated hepatic metastasis did not significantly improve overall survival, it did prolong substantially recurrence-free intervals compared to conventional medical therapy [11]. A longer recurrence-free period may allow for a break from cytotoxic chemotherapy, contributing to better quality of life. Meloni et al. reported that patients with hepatic metastases smaller than 2.5 cm had significantly longer survival after RFA compared to those with larger lesions [12]. The local recurrence rate after RFA for hepatic metastases from breast cancer has been reported to range from 14% to 50% [13]. In the present case, a 1.5 cm hepatic metastasis was treated with RFA, and no hepatic recurrence was detected for 3 years and 3 months. During this time, the patient was treated with eribulin mesilate or gemcitabine with minimal adverse effects, maintaining a good quality of life. While this is a single case, it suggests that RFA may be a useful option for similar cases and merits further investigation.

TNBC accounts for 10%-20% of invasive breast cancers and characterized to have poorer prognosis compared to other breast cancer subtypes [1]. PARP inhibitors such as olaparib and talazoparib have been approved for unresectable/recurrent breast cancer with germline *BRCA1* or *BRCA2* (*BRCA1/2*) mutations and without HER2 expression. More recently, immune checkpoint inhibitors such as pembrolizumab (monoclonal antibody against programed death-1 (PD-1)) and atezolizumab (monoclonal antibody against programed death ligand 1(PD-L1)) have become available for TNBC that is PD-L1-positive. However, only a minority of TNBC cases harbor these markers, approximately 15%-25% for *BRCA1/2* mutations [14] and about 20% for PD-L1 positivity [15]. Therefore, the majority of patients with small, solitary, or limited hepatic metastases from TNBC may be good candidates for RFA. In this case, the patient had wild-type *BRCA1/2* and was PD-L1-negative, making her ineligible for these newer targeted therapies.

## Conclusions

RFA may offer a prolonged period without hepatic recurrence in cases of small and solitary, or limited, hepatic metastases from TNBC. Maintaining quality of life is possible while metastatic lesions do not involve vital organs. However, as seen in this case, even with liver metastasis controlled, new metastases to other organs can still occur, making long-term survival unlikely.

PARP inhibitors and immune checkpoint inhibitors are potential treatment options for TNBC. However, these therapies are ineffective in tumors without BRCA1/2 mutations or PD-L1 expression. Further development of new targeted treatments for TNBC remains necessary.
